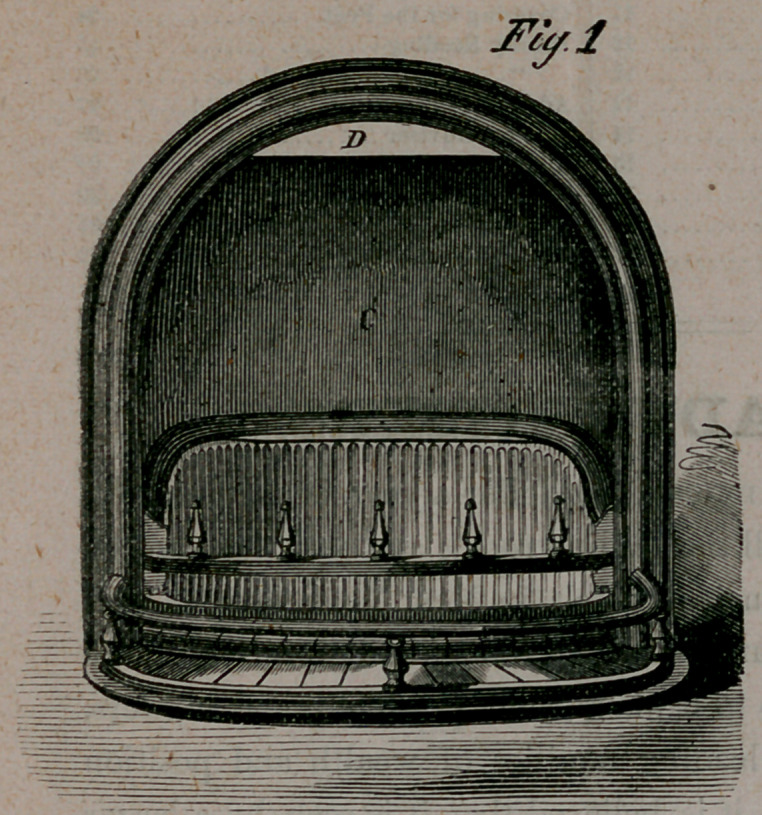# Fire on the Hearth

**Published:** 1875-02

**Authors:** 


DIXON'S PHILADELPHIA GRATE WORKS.

### FIRE ON THE HEARTH,

### *Or, Low-Down Grate.*

Fig. 1

A HARD COAL FIRE, burning brightly, flat on the hearth, on a level with the floor, warming the feet delightfully. with an oval fire-place nearly three feet across, with no visible blower, very little dust, and absolutely no gas, the ashes need removing but once a year, while by the extra heat, pure air direct from out doors is conveyed to an upper room, without the possibility of meeting any red-hot metallic surface, or with any corrupting surface whatever—it is simply pure air warmed. A Philadelphia correspondent says: "I have never known a day that a fire made in the morning was not equal to the day, no matter what the temperature was outside." A New England gentleman writes, after using the grate several years: "If there were no other grate to be had, a hundred times the price of it would not induce me to part with it." One of these grates can be set in an ordinary fire-place in half a day, costing from **$30** to **$150**, according to size and finish. They consume about the same amount of coal as an ordinary grate, and give out a softer, healthier and greater amount of heat, by at least one-fourth. The same house imports *French and English Grates, Fire Irons, Fenders, Screens, Andirons, Dog Grates*, besides having an extensive manufactory of their own of *Cooking Ranges, Warm Air Corrugated Cast Iron Furnaces and Parlor Grates*, for public and private buildings, with *Registers, Ventilators, etc.* They have recently patented the

### GAS LOG FIRE-PLACE,

Costing from **$12** to **$25**, and lasting a lifetime, resembling an old-fashioned fire-place piled up with hickory logs, all aglow with a brightly burning flame, supplied by a gas pipe, at a cost of ten cents an hour, with gas at $3.00 a thousand cubic feet. It is kindled in an instant, and extinguished as soon, without dust or smoke, or odor of gas, for this goes up the chimney. The cheeriness of a parlor thus warmed and enlivened, is incalculably preferable to the funereal gloominess of modern reception-rooms, with an atmosphere at once heavy, noisome, and oppressive.

Very recently the Franklin Institute of Philadelphia, the oldest in the country, has accorded to Thos. S. Dixon & Sons two Silver Medals for the Best Low-Down Grate, as an evidence of their hearty appreciation of its great practical value.

Address,

### THOS. S. DIXON & SONS,

### 1324 Chestnut Street, Philadelphia, Pa.

EASTERN AGENTS.—George E. Woodward, 191 Broadway, New York; Peck Sperry, New Haven, Ct.; D. S. Brooks & Sons, Hartford, Ct.; Murdock & Co., Boston, Mass.; O. M. & D. W. Nash, Portland, Me.

## Figures and Tables

**Fig.1. f1:**